# *b3galt6* Knock-Out Zebrafish Recapitulate β3GalT6-Deficiency Disorders in Human and Reveal a Trisaccharide Proteoglycan Linkage Region

**DOI:** 10.3389/fcell.2020.597857

**Published:** 2020-12-10

**Authors:** Sarah Delbaere, Adelbert De Clercq, Shuji Mizumoto, Fredrik Noborn, Jan Willem Bek, Lien Alluyn, Charlotte Gistelinck, Delfien Syx, Phil L. Salmon, Paul J. Coucke, Göran Larson, Shuhei Yamada, Andy Willaert, Fransiska Malfait

**Affiliations:** ^1^Department of Biomolecular Medicine, Center for Medical Genetics Ghent, Ghent University Hospital, Ghent University, Ghent, Belgium; ^2^Department of Pathobiochemistry, Faculty of Pharmacy, Meijo University, Nagoya, Japan; ^3^Department of Laboratory Medicine, Sahlgrenska Academy at the University of Gothenburg, Gothenburg, Sweden; ^4^Laboratory of Clinical Chemistry, Sahlgrenska University Hospital, Gothenburg, Sweden; ^5^Department of Orthopaedics and Sports Medicine, University of Washington, Seattle, WA, United States; ^6^Bruker MicroCT, Kontich, Belgium

**Keywords:** *b3galt6*, zebrafish, trisaccharide linkage region, proteoglycans, linkeropathies

## Abstract

Proteoglycans are structurally and functionally diverse biomacromolecules found abundantly on cell membranes and in the extracellular matrix. They consist of a core protein linked to glycosaminoglycan chains via a tetrasaccharide linkage region. Here, we show that CRISPR/Cas9-mediated *b3galt6* knock-out zebrafish, lacking galactosyltransferase II, which adds the third sugar in the linkage region, largely recapitulate the phenotypic abnormalities seen in human β3GalT6-deficiency disorders. These comprise craniofacial dysmorphism, generalized skeletal dysplasia, skin involvement and indications for muscle hypotonia. In-depth TEM analysis revealed disturbed collagen fibril organization as the most consistent ultrastructural characteristic throughout different affected tissues. Strikingly, despite a strong reduction in glycosaminoglycan content, as demonstrated by anion-exchange HPLC, subsequent LC-MS/MS analysis revealed a small amount of proteoglycans containing a unique linkage region consisting of only three sugars. This implies that formation of glycosaminoglycans with an immature linkage region is possible in a pathogenic context. Our study, therefore unveils a novel rescue mechanism for proteoglycan production in the absence of galactosyltransferase II, hereby opening new avenues for therapeutic intervention.

## Introduction

Proteoglycans (PGs) comprise a group of complex biomacromolecules, consisting of a core protein and one or multiple glycosaminoglycan (GAG) side chains, that are ubiquitously expressed in the extracellular matrix (ECM) and on cell surfaces and basement membranes in vertebrate and invertebrate species (Prydz and Dalen, 2000; Selleck, 2000; Couchman and Pataki, 2012; Lindahl et al., 2015). Besides their structural role, PGs act as mediators between the ECM and intracellular signaling pathways and contribute to development and tissue homeostasis, influencing a variety of cellular processes including cell fate determination, cell proliferation, migration, adhesion, differentiation, and survival (Malfait et al., 2013).

**FIGURE 1:**
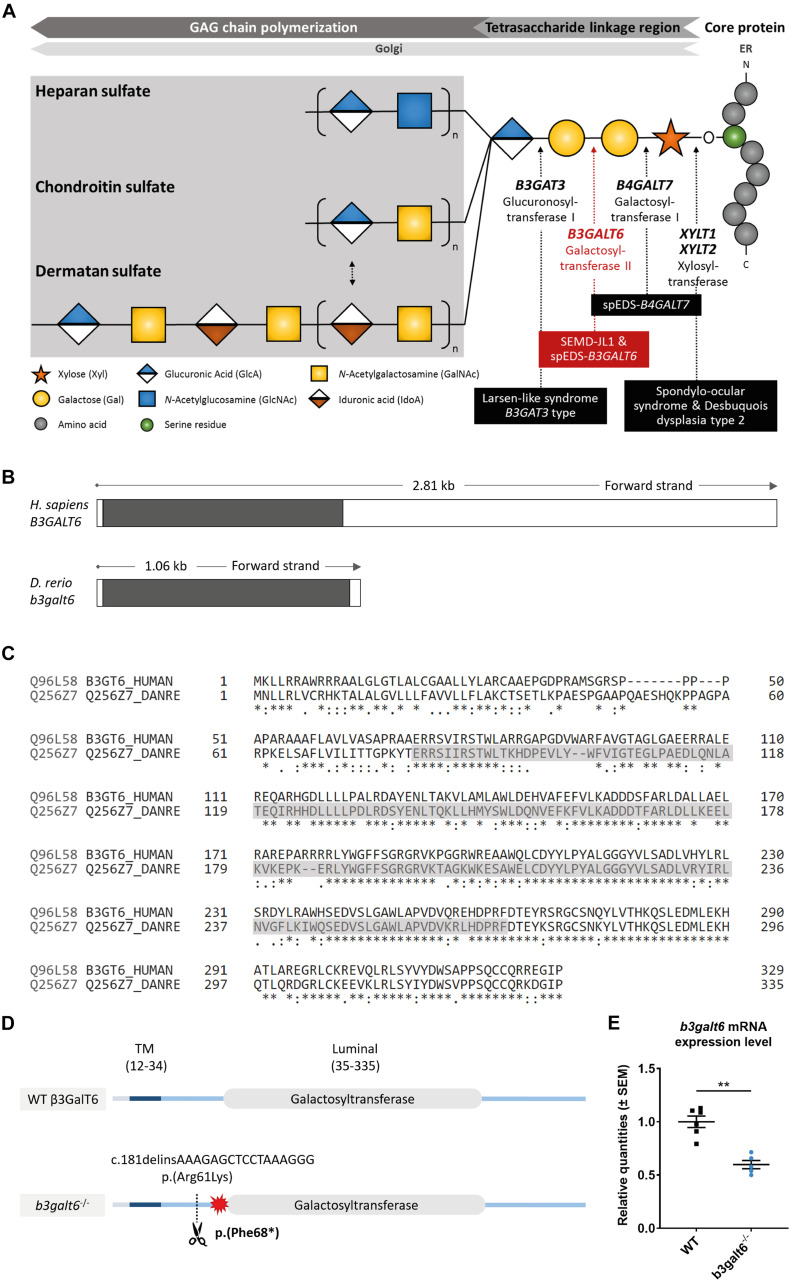
Schematic representation of the GAG biosynthesis and the evolutionary conservation of the zebrafish and human *b3galt6*/*B3GALT6* gene and B3galt6/β3GalT6 protein. **(A)** Simplified schematic representation of the PG linkage and GAGs biosynthesis. PG core proteins are synthesized in the endoplasmic reticulum and undergo further modification in the Golgi apparatus. First, a tetrasaccharide linkage region is synthesized that originates from the addition of a Xyl onto a serine residue of the core protein. Subsequent addition of two Gals and one GlcA residues completes this process. Depending on the addition of GlcNAc or GalNAc to the terminal GlcA residue of the linkage region, HS or CS/DS PGs will be formed, respectively. The elongation of the GAG chain continues by repeated addition of uronic acid and amino sugar residues. The GAG chain is then further modified by epimerization and sulfation (not shown). The main genes encoding for the enzymes involved in the linkage region biosynthesis are indicated in bold and biallelic mutations in these genes result in different linkeropathies. Disorders resulting from deficiency of the enzymes are indicated in black boxes. The deficient enzyme (β3GalT6) and the related disorder studied here are depicted in red (e.g., spEDS-*B3GALT6* and SEMD-JL1). **(B)** Schematic representation of the genomic structure of the *H. sapiens B3GALT6* (NM_080605.4) and *D. rerio b3galt6* (NM_001045225.1) genes. The white and black boxes represent the untranslated regions and the single *B3GALT6*/*b3galt6* exon, respectively. The zebrafish *b3galt6* gene resides on chromosome 11, spans a region of 1.06 kb and has not been duplicated during teleost evolution. **(C)** Amino acid (AA) sequence alignment (Clustal Omega) between *H. sapiens* β3GalT6 (Uniprot: Q96L58) and *D. rerio* B3galt6 (Uniprot: Q256Z7). The luminal galactosyltransferase domain is highlighted in gray. Conservation of AA sequences is shown below the alignment: “*” residues identical in all sequences in the alignment; “:” conserved substitutions; “.” semi-conserved substitutions; “space” no conservation. **(D)** Schematic representation of the cmg20 allele in *b3galt6* mutants. *b3galt6* consists of three parts, a cytosolic domain (AA 1-11), a transmembrane domain (TM, AA 12-34) and a luminal domain (AA 35-335). The location of the CRISPR/Cas9-generated indel variant is indicated by a scissor symbol and the resulting premature termination codon (PTC) is depicted by a red star. **(E)** RT-qPCR analysis of relative *b3galt6* mRNA expression levels in *b3galt6*^–/–^ adult zebrafish (0.597 ± 0.03919) (*n* = 5) compared to WT siblings (1 ± 0.05395) (*n* = 6). Data are expressed as mean ± SEM. A Mann-Whitney U test was used to determine significance, **P < 0.01.

The PG superfamily is, depending on the composition of the GAG chains, subdivided into heparan sulfate (HS) and chondroitin/dermatan sulfate (CS/DS) PGs. These GAG chains are linear polysaccharides, composed of a repeated disaccharide unit consisting of an uronic acid and amino sugar. HSPGs, such as perlecan, syndecan and glypican, consist of repeating glucuronic acid (GlcA)-*N*-acetyl-glucosamine (GlcNAc) disaccharides. CSPGs consist of repeating GlcA-*N*-acetyl-galactosamine (GalNAc) disaccharides. DS is a stereoisomer of CS, in which GlcA is epimerized to iduronic acid (IdoA). In mammalian tissues, CS and DS chains are often present as hybrid CS-DS chains on PGs (e.g. decorin, biglycan) (Sugahara et al., 2003). Tightly controlled modifications, such as epimerization and sulfation reactions, further increase the structural and functional diversity of the HS and CS/DS PGs (Kusche-Gullberg and Kjellen, 2003; Bui et al., 2010). GAG biosynthesis of both HSPGs and CS/DSPGs is initiated in the endoplasmic reticulum and Golgi apparatus by the formation of a common tetrasaccharide linkage region (glucuronic acid-galactose-galactose-xylose-*O*-) (GlcA-Gal-Gal-Xyl-*O*-) via *O*-glycosylation of a serine residue of the core protein (Figure 1A; Prydz and Dalen, 2000; Couchman and Pataki, 2012; Lindahl et al., 2015). The first step in the biosynthesis of this tetrasaccharide linkage region is catalyzed by xylosyltransferases (XylTs) encoded by the paralogs *XYLT1* [MIM 608124] and *XYLT2* [MIM 608125] (Gotting et al., 2000). Two Gal residues are subsequently added by, respectively, galactosyltransferase I (GalT-I or β4GalT7 encoded by *B4GALT7* [MIM 604327]) and galactosyltransferase II (GalT-II or β3GalT6 encoded by *B3GALT6* [MIM 615291]) (Almeida et al., 1999; Bai et al., 2001). Further addition of a GlcA by glucuronosyltransferase I (GlcAT-I encoded by *B3GAT3)* [MIM 606374] completes the formation of the linkage region (Kitagawa et al., 1998).

The cellular machinery required for GAG biosynthesis and modification is conserved over a broad range of eukaryotic organisms (Wen et al., 2014). The particular importance of a correct initiation of GAG synthesis is underscored by the identification of a series of severe, overlapping and multisystemic genetic human disorders that result from deficient activity of any of these “linkage-enzymes,” collectively coined as “linkeropathies” (Guo et al., 2013; Nakajima et al., 2013; Jones et al., 2015). The most frequently reported linkeropathy results from galactosyltransferase II deficiency, caused by biallelic pathogenic variants in *B3GALT6.* This leads to two clinically overlapping conditions, spondylodysplastic Ehlers-Danlos syndrome (EDS) (spEDS-*B3GALT6*) and spondyloepimetaphyseal dysplasia with joint laxity type I (SEMD-JL1) (Malfait et al., 2013; Nakajima et al., 2013), both of which are characterized by variable degrees of spondyloepimetaphyseal bone dysplasia (with postnatal growth restriction, bowing of long bones, hypo- or dysplasia of iliac and long bones, and vertebral body changes), kyphoscoliosis, bone fragility with spontaneous fractures, joint hypermobility in conjunction with joint contractures, hypotonia with delayed motor development, skin fragility, and craniofacial dysmorphisms, including midfacial hypoplasia and dysplastic teeth (Malfait et al., 2013, 2017; Nakajima et al., 2013). The severe and pleiotropic phenotype suggests a critical role for *B3GALT6* in the development and homeostasis of various connective tissues. Pathogenic variants in *B3GALT6* either result in mislocalization and/or reduced amounts of active galactosyltransferase II (Ritelli et al., 2015). Previous studies on several biallelic *B3GALT6* variants showed that they resulted in a barely detectable *in vitro* galactosyltransferase II enzymatic activity, but intriguingly HS and CS/DS GAG synthesis was partially preserved *in cellulo* (Van Damme et al., 2018). While some variants may have a greater deleterious effect on the *in vitro* than on the *in cellulo* enzymatic activity, it is currently unknown whether there are rescue mechanisms at play to partially compensate for the galactosyltransferase II activity.

In recent years, zebrafish have emerged as an excellent tool to model disease and to increase our understanding of developmental processes and disease mechanisms. Zebrafish share 70% of their genes with humans (Santoriello and Zon, 2012) and the advantages of their use are reflected by their large breed size, short lifecycle and low husbandry costs (Dooley and Zon, 2000). Zebrafish has proven to be a relevant genetic model for the study of PG biosynthesis since many enzymes involved in this process are strongly conserved between human and zebrafish (Eames et al., 2010), and a number of zebrafish models for defective PG synthesis (including *fam20b^b1125^, xylt1^b1189^, or b3gat3^hi307^*), provided novel knowledge on PG function in bone and cartilage formation (Amsterdam et al., 2004; Nissen et al., 2006; Eames et al., 2010, 2011).

In this study, we generated *b3galt6* knock-out (KO) zebrafish as a model for spEDS-*B3GALT6* and SEMD-JL1. These models were used to investigate the structure and amount of different GAG disaccharides and the PG linkage region composition in these disorders. Furthermore, in-depth phenotypic characterization of *b3galt6*^–/–^ zebrafish was done to reveal to which extent key features of the human disorders are recapitulated and to further elucidate the function of galactosyltransferase II in vertebrate musculoskeletal development.

## Results

### CRISPR/Cas9-Mediated *b3galt6* Knock-Out in Zebrafish Leads to Reduced HS and CS/DS Glycosaminoglycan Concentrations in Bone, Muscle, and Skin

Similar to its human ortholog, zebrafish *b3galt6* (NM_001045225.1) is a single-exon gene (Figure 1B). Human and zebrafish galactosyltransferase II (β3GalT6/B3galt6) protein sequence display 82.5% amino acid (AA) similarity, with the highest AA conservation in the luminal domain (Figure 1C).

In order to investigate the consequences of zebrafish B3galt6-deficiency on GAG biosynthesis, we generated two *b3galt6* KO zebrafish models using CRISPR/Cas9 gene editing. The first KO model carries the c.181delinsAAAGAGCTCCTAAAGGG indel mutation (cmg20 allele), leading to a premature termination codon (PTC) [p.(Phe68^∗^)], located upstream to the catalytic domain (Figure 1D). RT-qPCR showed a 40.3% reduction of *b3galt6 mRNA* expression in homozygous adult *b3galt6* KO zebrafish compared to wild-type (WT) siblings (Figure 1E). For the remainder of this manuscript, this model will be referred to as *b3galt6*^–/–^. The second KO model carries a four base pair deletion (c.398_401del) (cmg22 allele), predicted to generate a PTC [p.(Asn139^∗^)] in the catalytic domain (NM_001045225.1). This mutant was solely used for validation purposes (Supplementary Figure S1). Both *b3galt6* KO zebrafish were produced at an expected Mendelian ratio and survived into adulthood. Unfortunately, commercial antibodies, which were only available against human β3GalT6, did not work in our hands and precluded the investigation of zebrafish B3galt6 protein levels and localization.

To examine the activity of B3galt6 in different *b3galt6*^–/–^ tissues, concentrations of CS, DS, and HS disaccharides extracted from bone, muscle and skin from WT and *b3galt6*^–/–^ zebrafish were quantified using a combination of enzymatic digestion and anion-exchange HPLC (Supplementary Figures S2–S5 and Supplementary Tables S1–S4). Overall, a generalized decrease in HS, CS and DS concentration was detected in all examined tissues of *b3galt6*^–/–^ zebrafish. In the GAG fractions prepared from bone, the disaccharide concentration of the CS/DS and CS moieties was significantly decreased by, respectively, 55 and 64%, compared to WT zebrafish (Table 1 and Supplementary Tables S1, S2). DS moieties were undetectable in bone of *b3galt6*^–/–^ zebrafish, whereas their concentration was 50.5 pmol/mg protein in bone of WT zebrafish (Table 1 and Supplementary Table S3). A non-significant decrease in the disaccharide concentration of the HS moiety was observed in *b3galt6*^–/–^ mutant bone (Table 1 and Supplementary Table S4), compared to WT. In GAG fractions prepared from *b3galt6*^–/–^ muscle, CS/DS, CS, and DS disaccharide moieties were generally lower than in WT muscle (Table 1 and Supplementary Tables S1–S3). Due to the low concentrations close to the detection limit, a significant reduction could only be detected for the DS moiety (72% reduction) (Table 1 and Supplementary Table S3). In addition, a significant decrease in HS disaccharide moieties by 56% was noted in *b3galt6*^–/–^ muscle compared to WT muscle (Table 1 and Supplementary Table S4). In the GAG fractions prepared from *b3galt6*^–^*^/^*^–^ skin, the concentration of all disaccharide moieties was significantly reduced compared to WT (Table 1 and Supplementary Tables S1–S4). The disaccharide concentration of the CS/DS, CS, DS and HS moieties were decreased by, respectively, 77, 73, 55, and 62% compared to WT (Table 1 and Supplementary Tables S1–S4). No altered sulfation patterns were observed in disaccharide moieties when comparing unique GAG chains between WT and *b3galt6*^–/–^ zebrafish samples (Supplementary Tables S1–S4).

**TABLE 1:** Concentrations of GAG disaccharides in wild-type and *b3galt6*^–/–^ zebrafish tissues.

	*pmol/mg protein (Average* ± *SE)*			
	
	CS/DS	CS	DS	HS
**Bone**				
Wild-type	774.2 ± 19.9	728.4 ± 148.1	50.5 ± 8.1	178.6 ± 19.8
*b3galt6*^–^*^/^*^–^	346.1 ± 20.0*	279.9 ± 4.8**	N.D.	137.1 ± 39.7^ns^
**Muscle**				
Wild-type	9.3 ± 0.4	2.1 ± 0.4	14.5 ± 1.1	30.8 ± 1.2
*b3galt6*^–^*^/^*^–^	5.7 ± 3.1^ns^	N.D.	4.1 ± 1.0*	13.5 ± 4.0**
**Skin**				
Wild-type	236.2 ± 26.5	175.1 ± 25.3	75.9 ± 7.9	55.1 ± 4.5
*b3galt6*^–^*^/^*^–^	55.4 ± 15.2*	47.9 ± 13.5**	33.8 ± 6.3**	21.0 ± 2.8**

Student’s t-test (vs. wild-type) (n = 3), N.D., not detected (< 1 pmol/mg protein), *P < 0.01, **P < 0.05, ns: not significant, SE: standard error.

Despite the strong reduction in disaccharide concentrations observed in *b3galt6*^–/–^ zebrafish, low amounts of GAGs were still produced in the mutants. Similar results were obtained for the second *b3galt6* KO zebrafish (cmg22 allele) (Supplementary Table S5). RT-qPCR revealed no significant upregulation of other galactosyltransferase(s) member(s) from the *b3galt* family (*b3galt1a*, *b3galt2*, *b3galt4*, *b3galnt2*) or of other linkage-enzymes (*xylt1*, *xylt2*, *b4galt7* and *b3gat3*) (Supplementary Figure S6), suggesting that altered expression of these genes did not compensate for the reduced *b3galt6* mRNA levels.

### B3galt6-Deficiency Leads to the Biosynthesis of a Non-canonical (GlcA-Gal-Xyl-*O*-) Trisaccharide Linkage Region in Zebrafish

**FIGURE 2:**
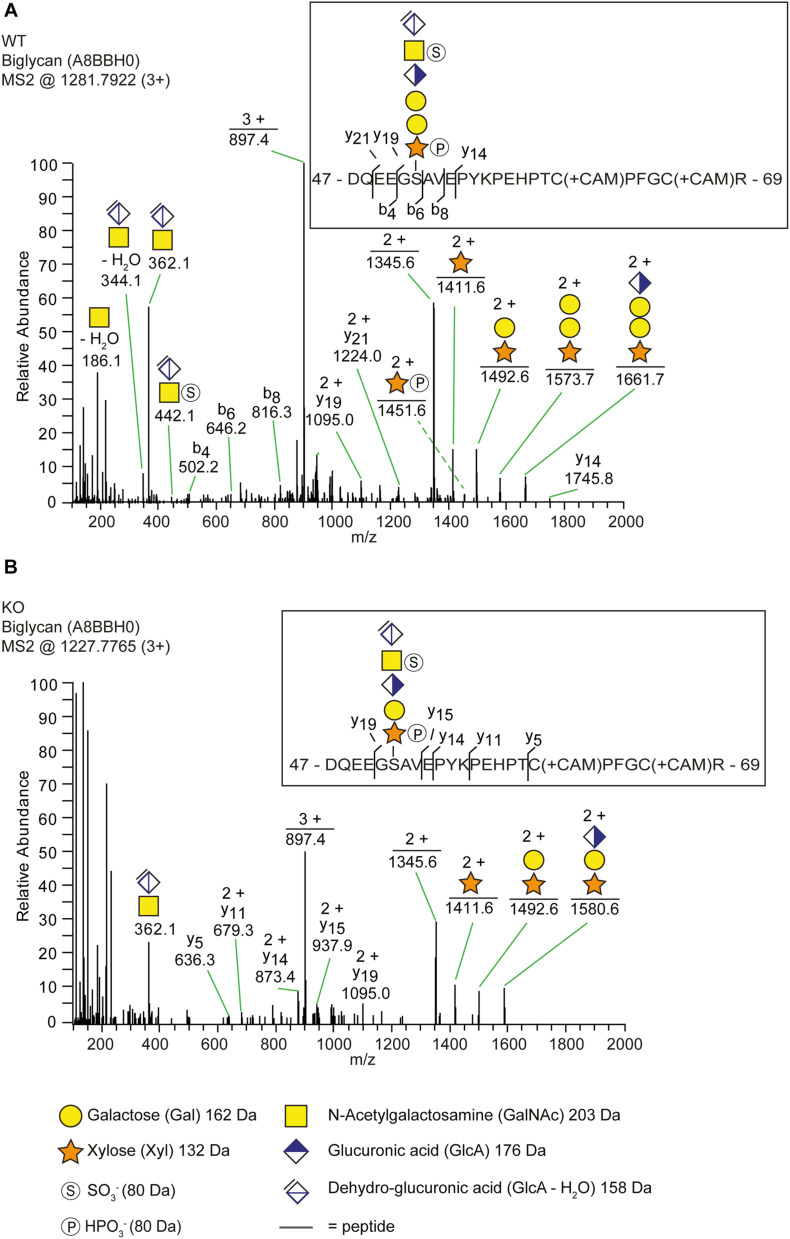
The identification of a trisaccharide linkage region in the *b3galt6*^–/–^ zebrafish. **(A,B)** MS2 fragment mass spectra of CS-glycopeptides derived from biglycan (UniProt: A8BBH0) showed different linkage regions in WT compared to the *b3galt6*^–/–^ zebrafish. **(A)** The peptide (DQEEGSAVEPYKPEHPTCPFGCR) in WT zebrafish was modified with a residual hexasaccharide structure, corresponding to the canonical tetrasaccharide linkage region (*m/z* 1281.79; 3+). The hexasaccharide structure was modified with one sulfate group (SO ${}_{3}^{-}$) at the subterminal GalNAc residue and one phosphate group (HPO${}_{3}^{-}$) at the xylose residue (Supplementary Figure S7). **(B)** The peptide (DQEEGSAVEPYKPEHPTCPFGCR) in *b3galt6*^–/–^ zebrafish was modified with a residual pentasaccharide structure, corresponding to a non-canonical trisaccharide linkage region (*m/z* 1227.78; 3+). The pentasaccharide structure was modified with two phosphate/sulfate groups. Their exact positioning and identity could not be determined due to low intensities of corresponding ion peaks, and the positions are therefore assigned in analogy of the WT structure.

In order to explain why *b3galt6*^–/–^ zebrafish can still produce GAG chains on PG core proteins, we investigated the composition of the linkage region. To this purpose, whole zebrafish extracts were enriched for all PGs, their GAG chains depolymerized with bacterial lyases, the core proteins digested with trypsin and the remaining glycopeptides subjected to nano-scale liquid chromatography-tandem mass spectrometry (nLC-MS/MS) using a recently developed glycoproteomic approach (Noborn et al., 2015; Persson et al., 2019). Potential structural differences in PG linkage regions in WT versus *b3galt6*^–/–^ zebrafish are thus elucidated through the combined identities of peptide backbones and GAG linkage region structures. The glycoproteomic analysis identified two CS-glycopeptides derived from biglycan (UniProt: A8BBH0) in both the WT and *b3galt6*^–/–^ zebrafish. These CS-glycopeptides displayed different linkage region structures in WT compared with *b3galt6*^–/–^ zebrafish (Figure 2). While the peptide in the WT zebrafish was modified with a hexasaccharide structure (Figure 2A), the peptide in the *b3galt6*^–/–^ zebrafish was modified with a pentasaccharide structure (Figure 2B), supporting the hypothesis that *b3galt6*^–/–^ zebrafish produces a non-canonical trisaccharide linkage region. The precursor ion (m/z 1281.7922; 3+) in the WT zebrafish equated to a mass of a peptide (DQEEGSAVEPYKPEHPTCPFGCR) modified with a hexasaccharide structure, with the peptide containing two carbamidomethyl (CAM) modifications. Furthermore, the hexasaccharide structure was modified with one sulfate group (SO ${}_{3}^{-}$) on the subterminal GalNAc residue [GlcAGalNAc-H_2_O + SO_3_ + H]^+^, and one phosphate group (HPO${}_{3}^{-}$) on the Xyl residue (Supplementary Figure S7). In contrast, the precursor ion (*m/z* 1227.7765; 3+) in the *b3galt6*^–/–^ zebrafish equated to a mass of the same peptide but modified with a pentasaccharide structure, with two phosphate/sulfate groups, with the peptide containing two CAM modifications. The difference in mass between the WT and *b3galt6*^–/–^ precursor ions corresponds to 162.08 Da, equating to the mass of a hexose (galactose) residue (162 Da). Additional HS- and CS-glycopeptides with the expected tetrasaccharide linkage region were found in the WT. In the *b3galt6*^–/–^, no HS-modified structures were identified but additional CS-glycopeptides with the non-canonical trisaccharide linkage region were found. Supplementary Table S6 shows a complete list of all glycopeptides identified in both WT and *b3galt6*^–/–^ zebrafish. PGs harboring a tetrasaccharide linkage region were never detected in the *b3galt6*^–/–^ adult zebrafish samples and no trisaccharide linkage region was detected in WT zebrafish samples.

These observations suggest that B3GAT3/GlcAT-I can transfer a GlcA residue not only to Gal-Gal-Xyl, but also to a Gal-Xyl substrate. This was confirmed by an *in vitro* GlcAT-I assay, which showed that GlcAT-I is indeed capable of adding a GlcA to a Gal-Xyl-*p*-nitrophynyl, which was chemically synthesized, thus to a substrate ending with a single galactose (Supplementary Figure S8).

### *b3galt6*^–/–^ Zebrafish Show Early Onset, Progressive Morphological Abnormalities and Delay of Cartilage and Bone Formation, Reminiscent of Human spEDS-*B3GALT6*

One of the clinical hallmarks of human spEDS-*B3GALT6* is the pronounced skeletal involvement with the presence of spondyloepimetaphyseal dysplasia with postnatal growth restriction, bowing of long bones, hypoplasia of iliac bones, femoral head dysplasia, vertebral body changes, kyphoscoliosis and bone fragility with spontaneous fractures (Table 2). To evaluate the development of cartilage and bone associated with B3galt6-deficiency in zebrafish, phenotypic characterization of *b3galt6*^–^*^/^*^–^ zebrafish was performed in juvenile (20 dpf, days post fertilization) and adult stages (4 months).

**TABLE 2:** Clinical features and ultrastructural and biochemical abnormalities observed in spEDS-*B3GALT6* and SEMD-JL1 individuals compared to the *b3galt6*^–/–^ zebrafish model.

		Human		Zebrafish
			
		spEDS-*B3GALT6*	SEMD-JL1	*b3galt6*^–/–^ mutant
General morphology				
	Short stature	22/30	11/14	Reduced body length
	Craniofacial dysmorphisms (e.g., midfacial hypoplasia, micrognatia)	25/30	12/14	Deformed, small and round head with shorter parietal/occipital region and underdeveloped olfactory region
	Dental abnormalities	14/30	n.a.	Smaller teeth
Musculoskeletal involvement				
	Bowing of limbs, metaphyseal widening or vertebral body changes	20/30	14/14	Deformities of the fin skeleton
	Bone fragility with spontaneous fractures	19/30	0/3	Extra intramembranous bone, lower degree of collagen type I fibril organization and higher TMD after correction for smaller size
	Joint contractures (especially hands)	23/30	5/14	Pectoral fins point upwards
	Joint hypermobility	26/30	6/14	n.a.
	Kyphoscoliosis	23/30	13/14	Kyphosis and scoliosis
	Muscle hypotonia	18/30	1/14	Reduced critical swimming speed and endurance
Skin features				
	Skin hyperextensibility	19/30	2/14	n.a.
Biochemical findings				
	Reduced GAG synthesis	9/9	4/4 (3/4; <HS but >CS/DS)	Reduced GAG production
Ultrastructural findings				
	Loosely packed collagen fibrils	1/1	n.a.	Disturbed collagen fibril organization in bone and loosely packed collagen fibrils in the dermis

*n.a., not available; GAG, glycosaminoglycan. The columns “spEDS-B3GALT6” and “SEMD-JL1” summarize the most important clinical features of all hitherto reported patients for whom this data was available (Van Damme et al., 2018; Caraffi et al., 2019).*

**FIGURE 3:**
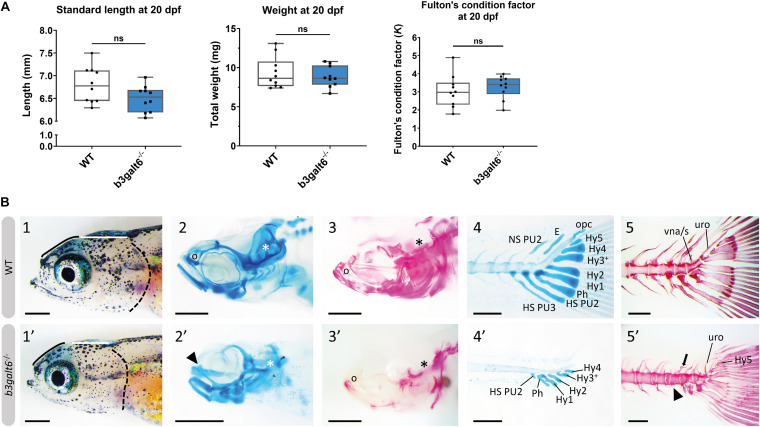
Developmental delay and morphological abnormalities in 20 dpf *b3galt6*^–/–^ zebrafish. **(A)** Weight, standard length and Fulton’s condition factor of WT and *b3galt6*^–/–^ zebrafish larvae at 20 dpf (*n* = 10). **(B)** External phenotype of the head of juvenile WT and *b3galt6*^–/–^ sibling zebrafish. All images are oriented anterior to the left, posterior to the right, dorsal to the top and ventral to the bottom. **(1-1’)** *b3galt6*^–/–^ zebrafish display a rounder frontal region (black line), shorter parietal/occipital region (white horizontal line) and a malformed posterior edge of the opercular apparatus (dotted black line), compared to WT control. **(2-2’)** Representative images of cartilage AB staining of the head. The olfactory region (indicated by “o” in the WT and arrowhead in the *b3galt6*^–/–^ picture) and the otic region (indicated by white asterisk) show delayed development of cartilage elements in *b3galt6*^–/–^ zebrafish as compared to WT. **(3-3’)** Representative images of AR mineral staining of the head. The olfactory region (indicated by “o”) and the otic region (indicated by asterisk) show severely delayed mineralization in *b3galt6*^–/–^ zebrafish larvae as compared to WT. **(4-4’)** Representative images of AB staining of the caudal fin. The modified associated elements show developmental delay in *b3galt6*^–/–^ zebrafish as compared to WT. E, epural; HS PU2, haemal spine of preural 2; HS PU3, haemal spine of preural 3; Hy1–5, hypural 1–5; NS PU2, neural spine preural 2; opc, opistural cartilage; Ph, parhypural **(5-5’)** Representative images of AR mineral staining of the caudal fin. Caudal vertebrae and their associated elements show no delay in ossification. Malformation or absence of caudal fin associated elements (block arrow indicates a neural arch; arrowhead indicates a missing haemal arch), and malformation of uroneural (uro) and vestigial neural arches and spines (vna/s) are observed in *b3galt6*^–/–^ zebrafish; hypurals have a bent appearance. Scale bars: images of the head 500 μm, images of the caudal fin 200 μm.

At 20 dpf, the average weight (W), standard length (SL) and Fulton’s condition factor (*K*), which is used as a measure for the relative proportion of SL to W (Nash et al., 2006), were not significantly different between *b3galt6*^–^*^/^*^–^ and WT siblings (Figure 3A), although for SL there was a trend toward smaller mutants (*P* = 0.0640). Several externally visible morphological anomalies could be observed in 20 dpf *b3galt6*^–^*^/^*^–^ zebrafish as compared to their WT siblings, including a shorter, more compact head, a shorter and rounder frontal region, a shorter parietal/occipital region and a malformed posterior edge of the opercular apparatus (Figure 3B). Alcian blue (AB) cartilage staining showed no morphological abnormalities, but developmental delay of the head and pectoral and caudal fin endoskeleton was noted (Figure 3B and Supplementary Table S7), with cartilage elements being absent or in early development in *b3galt6* KO, while these structures were already fully developed in WT sibling zebrafish. Alizarin red (AR) mineral staining showed delayed mineralization in all regions of the head, the pectoral fin girdle and the caudal fin (Figure 3B and Supplementary Table S8), further confirming developmental retardation. In addition, malformations of different mineralized bones, such as the parasphenoid, branchiostegal rays, supracleithrum, and the subopercle bone could be detected in the head of *b3galt6*^–/–^ zebrafish larvae (Supplementary Figure S9). The vertebral column, i.e., the vertebral centra and their associated elements (neural and haemal arches), did not show delayed mineralization in *b3galt6*^–/–^ zebrafish larvae, but malformation of the associated elements and fusions of preural vertebrae were observed at a much higher frequency in the *b3galt6*^–/^*^–^* mutants. Kyphosis, scoliosis and lordosis were also more frequently observed and pronounced in *b3galt6*^–/^*^–^* larvae compared to WT siblings (Supplementary Figure S10 and Supplementary Table S8). A similar phenotype was observed in the second *b3galt6* KO model (cmg22 allele), confirming the specificity of the displayed phenotype (Supplementary Figure S11).

**FIGURE 4:**
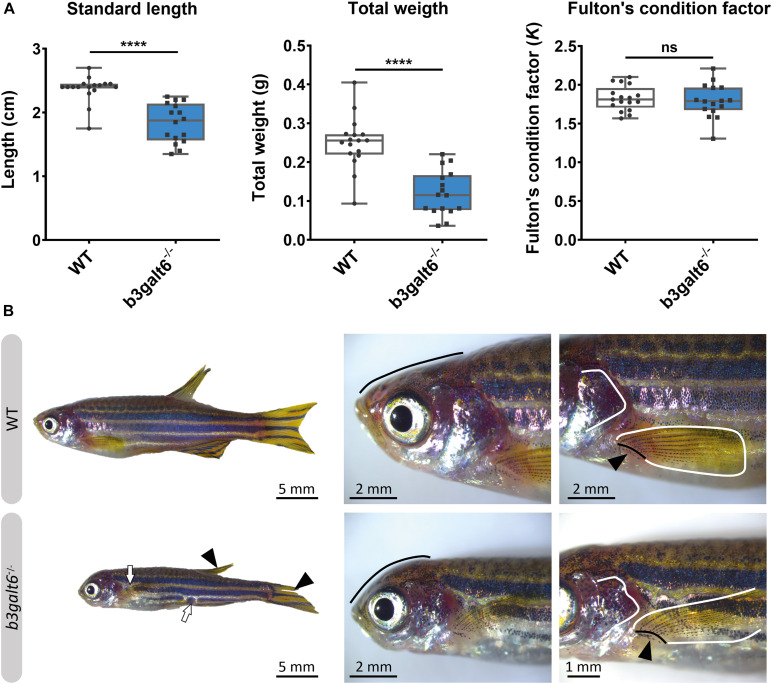
Characterization of the external phenotype of adult *b3galt6*^–/–^ zebrafish. **(A)** *b3galt6*^–/–^ zebrafish (*n* = 16) had a significantly shorter standard length (SL) compared to their WT siblings (*n* = 17). The weight (W) was significantly lower in the mutant zebrafish. The Fulton’s condition factor [*K* = 100*(W/SL^3^)] was not significantly different between *b3galt6*^–/–^ zebrafish and WT siblings. Data are expressed as box plots with min-to-max whiskers on which each individual data point is plotted. An unpaired *t*-test with Welch’s correction was used to determine significance. ****P < 0.0001, ns: not significant. **(B)** Adult *b3galt6*^–/–^ zebrafish exhibit morphological abnormalities compared to their WT siblings; *b3galt6*^–/–^ zebrafish display interruptions of the blue horizontal stripes (white arrows), smaller fins (arrowhead), aberrations of the head shape (black line), of the shape of the opercular apparatus (white line indicates the posterior edge) and of the pectoral fin (black line and arrow indicate the fin rays at the base of the fin, white line shows the outline of the fin).

At the adult stage (4 months), *b3galt6*^–^**^/^***^–^* zebrafish had a significantly reduced average SL (1.834 ± 0.077 cm), and W (0.1196 ± 0.014 g), when compared to their WT siblings (2.371 ± 0.049 cm, and 0.2497 ± 0.016 g, respectively), while the Fulton’s condition factor (*K*) indicated that SL and W were normally proportioned to each other (Figure 4A). A similar phenotype was observed in the second *b3galt6* KO model (cmg22 allele), confirming the specificity of the displayed phenotype (Supplementary Figure S1).

The head malformations, which were already observed in 20 dpf *b3galt6*^–/^*^–^* zebrafish, showed further progression, with a more pronounced smaller, rounded head and an abnormal compaction of the opercular apparatus (Figure 4B). In addition, a reduced surface area of the fins, with a crenelated postero-distal fin edge was observed in the mutant animals. The pectoral fins were pointing in an abnormal upwards angle (Figure 4B). In addition, an altered pigmentation pattern was observed, mainly on the ventral part of the body where the dark stripes showed interruptions (Figure 4B).

**FIGURE 5:**
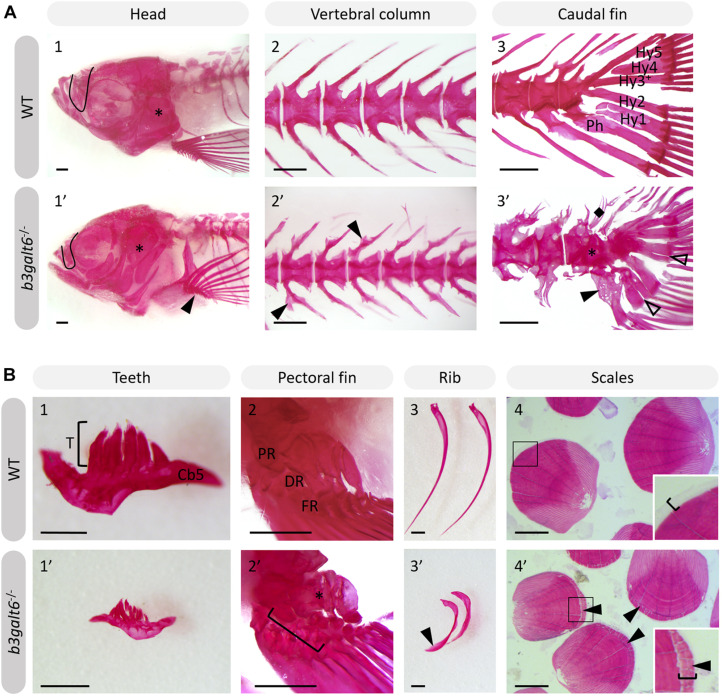
Alizarin red stained mineralized bone in WT and *b3galt6*^–/–^ adult zebrafish. The mineralized bones in the skeleton were studied in 4 months old WT and mutant zebrafish after AR mineral staining. **(A)** *b3galt6*^–/–^ zebrafish display malformation of the cranium, vertebral column and caudal fin. **(A1-1’)** The cranium shows an underdeveloped olfactory region (black line) and a smaller compacted opercular bone (asterisk). **(A2-2’)** The vertebral column shows extra intramembranous outgrowths located where the distal tip of the neural and haemal arches fuse to a neural and haemal spine, respectively (arrowheads). **(A3-3’)** In the caudal fin fusion of preural vertebrae is observed (asterisk). Associated elements show intramembranous outgrowths (arrowhead) and the presence of extra bony elements (diamond). The distal tip of the hypurals is sometimes split (transparent arrowhead). Structures indicated in the WT caudal fin: Hy1-5, hypural 1-5, Ph, parhypural. **(B)** Details of mineralized structures in *b3galt6*^–/–^ zebrafish. **(B1-1’)** The fifth ceratobranchial arch and its teeth are smaller in KO zebrafish. **(B2-2’)** The proximal (PR) and distal radials (DR) have irregular shapes in mutant zebrafish. Proximal (asterisk) and distal radials are sometimes fused. The bracket indicates a fusion between a proximal and distal radial, and the first fin ray (FR). **(B3-3’)** Ribs also show extra intramembranous bone (black arrowhead). **(B4-4’)** More mineralization was observed at the growth zone (bracket) of the scales in mutant zebrafish (arrowhead). Scale bars = 500 μm.

Further progression of the larval phenotype was also confirmed by AR staining of the mineralized skeleton in adult (4 months) *b3galt6*^–/^*^–^* zebrafish. The olfactory region was underdeveloped (Figure 5A), the cranial roof bones and sutures were deformed (Supplementary Figure S12) and the opercular apparatus was smaller and compacted in KO zebrafish (Figure 5A and Supplementary Figure S12). The fifth ceratobranchial arch and its attached teeth were smaller in *b3galt6*^–/^*^–^* zebrafish (Figure 5B). In the pectoral fins of *b3galt6*^–/^*^–^* zebrafish the radials were smaller, had an irregular shape, and were often fused (Figure 5B). The autocentra, i.e., the inner layer of the vertebral centra in the vertebral column and caudal fin (Arratia and Schultze, 1992) of KO zebrafish had a normal hourglass shape, but the associated elements often showed presence of extra intramembranous bone (Figure 5 and Supplementary Figure S12) and extra bony elements (Figure 5A). Fusions were observed in preural vertebrae (Figure 5A and Supplementary Figures S12B,C). Lordosis and scoliosis was variably observed in the caudal region of the vertebral column of *b3galt6*^–/^*^–^* mutant zebrafish (Supplementary Figures S12B,C). Extra mineralization was observed at the edge (growth zone) of *b3galt6*^–/^*^–^* scales (Figure 5B). Notably, variation in the severity of the skeletal malformations in *b3galt6*^–/^*^–^* zebrafish was observed.

**FIGURE 6:**
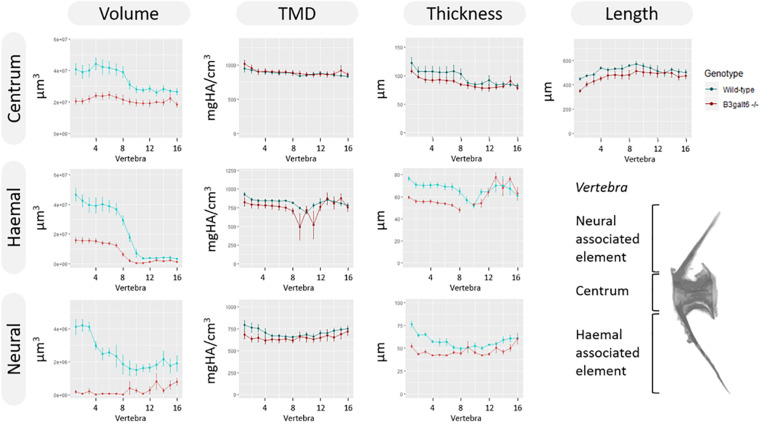
μCT analysis of adult WT and *b3galt6*^–/–^ zebrafish. Quantitative μCT analysis of the vertebral column in five mutants vs. five WT zebrafish at the age of 4 months. The bone volume, tissue mineral density (TMD) and bone thickness were calculated for three parts of each vertebra: the vertebral centrum, the neural associated element (neural arch and neural spine) and the haemal associated element (haemal arch and haemal spine). Plots associated with a significant difference are colored in a lighter coloring scheme. The *X*-axis represents each vertebra along the anterior-posterior axis. The volume is significantly reduced for each part of the vertebra in *b3galt6*^–/–^ zebrafish (*P* < 0.01, *P* < 0.001, and *P* < 0.000001, respectively). The thickness of the haemal (*P* < 0.01) and neural (*P* = 0.001) associated element is also significantly reduced in the mutants whereas the centrum thickness is not significantly reduced in *b3galt6*^–/–^ zebrafish (*P* = 0.12). TMD is not significantly different between *b3galt6*^–/–^ zebrafish and WT siblings when not normalized for standard length (*P* > 0.2). Data are presented as mean ± SEM. TMD, tissue mineral density; HA, hydroxyapatite.

Subsequent quantitative μCT analysis of 4 months old zebrafish revealed multifaceted effects of B3galt6-deficiency on vertebral morphology and mineralization. Most notably, the volume of vertebral centrum, haemal and neural associated elements was significantly reduced throughout the entire vertebral column in *b3galt6*^–/^*^–^* zebrafish (*P* < 0.01), with more pronounced differences in the abdominal region (Figure 6). Tissue mineral density (TMD) was not significantly different in the *b3galt6*^–/^*^–^* vertebral bodies compared to WT siblings. However, TMD in zebrafish is proportionally related to standard length (Parichy et al., 2009; McMenamin et al., 2016), and *b3galt6*^–/–^ zebrafish exhibited a significantly reduced body size compared to the WT siblings. We therefore normalized the μCT data to standard length using the normalization procedure outlined by Hur et al. (2017). In this procedure, phenotypic data in WT sibling controls was scaled using the mean standard length of *b3galt6*^–/–^ zebrafish as the reference length. Remarkably, after normalization, a significant increase in TMD of centrum, haemal and neural associated elements was noted in *b3galt6*^–/–^ zebrafish compared to WT controls (*P* < 0.001, *P* < 0.01, and *P* < 0.01, respectively) (Supplementary Figure S13).

### *b3galt6* Knock-Out Zebrafish Display a Disturbed Organization of Type I Collagen Fibrils in Skin, Bone, and Intervertebral Space

**FIGURE 7:**
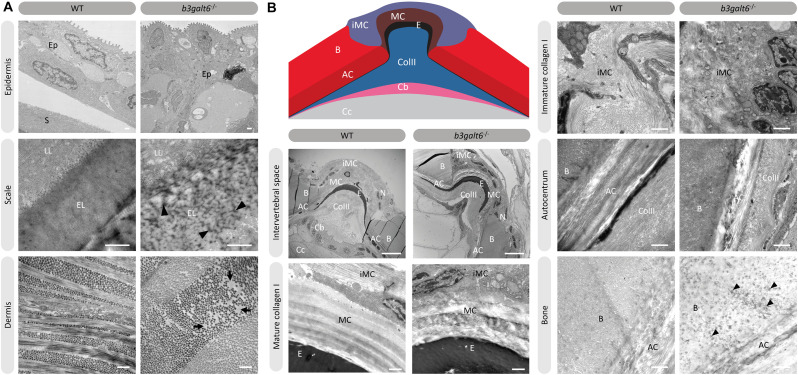
Collagen fibrillar architecture in 5 months old adult zebrafish. **(A)** Skin from 5 months old WT and *b3galt6*^–/–^ adult zebrafish. The epidermis (Ep), the outer layer of the skin covering the scales (S) of zebrafish, appears thicker in *b3galt6*^–/–^ zebrafish compared to the epidermis of WT zebrafish. A mature zebrafish scale consists of several layers. The limiting layer of the scale (LL) is rich in fine granules that in some areas are linearly arranged, forming dense layers. This matrix contrasts with that of the external layer(s) (EL) below. Structures resembling extrafibrillar crystals (arrowhead) are noted in the external layer of the mutant scale, which are rare in WT zebrafish sections. Upon investigation of the dermis of *b3galt6*^–/–^ mutants, increased interfibrillar spaces are noted (arrows), which are absent in the dermis of WT adults. Scale bars = 500 nm. **(B)** Schematic representation and TEM images of the zebrafish intervertebral space. Schematic representation of the zebrafish intervertebral space. AC, autocentrum; B, bone; Cb, chordoblasts; Cc, chordocytes; ColII, type II collagen; E, elastin; iMC, immature collagen type I layer; MC, mature collagen type I layer. Starting from the central body axis, several recognizable structures are present in the intervertebral space along a proximo-distal axis: (i) notochord tissue consisting of chordocytes and chordoblasts, (ii) the notochord sheath consisting of a type II collagen layer and an external elastic membrane, (iii) and a connective tissue ligament consistent of a mature and an immature type I collagen layer. The mature type I collagen layer is organized in a typical plywood-like pattern and is continuous with the inner layer of the vertebral bone, i.e., the autocentrum, which also shows a plywood-like organization. The immature type I collagen layers consist of loose collagen fibers connecting the outer layer of vertebral bones. Notice the higher number of nuclei (N) in the *b3galt6*^–/–^ zebrafish at the intervertebral space. Mature collagen (MC) displays a typical plywood-like organization, in WT zebrafish but not in comparable regions for *b3galt6*^–/–^ mutant zebrafish. Cross section and longitudinal type I collagen regions are less clearly demarcated and smaller in immature collagen (iMC) from *b3galt6*^–/–^ zebrafish compared to WT siblings. It is more difficult to pinpoint the different layers of the autocentrum (AC) in the KO compared to WT zebrafish. Bone (B) from the *b3galt6*^–/–^ zebrafish shows electron dense structures (arrowhead). Scale bars = 10 μm for the intervertebral space and 1 μm for the other images.

Transmission electron microscopy (TEM) studies on the dermis of spEDS-*B3GALT6* patients revealed an abnormal collagen fibril architecture with loosely packed collagen fibrils of variable size and shape (Malfait et al., 2013). We therefore studied the effect of loss of galactosyltransferase II activity on collagen fibril architecture. TEM sections of skin biopsies of *b3galt6*^–/–^ zebrafish showed an abnormal dermal collagen fibril architecture characterized by loosely packed collagen fibrils and larger and more interfibrillar spaces compared to WT siblings (Figure 7A). In addition, at the level of the scales a thicker epidermal layer was observed and electron dense structures were present in the interfibrillar spaces (Figure 7A). Next, we examined the intervertebral region and adjacent vertebral centra in the vertebral column of 5 months old zebrafish by means of TEM. In general, the type I collagen fibers in *b3galt6*^–^*^/^*^–^ zebrafish displayed a lower degree of organization (Figure 7B). This was observed in immature collagen layers, deposited by osteoblasts in the outer edges of the intervertebral space, but also in the mature type I collagen layers of the intervertebral ligament and in the autocentrum, the inner layer of the vertebral bone, where a lack of the typical plywood-like organization was seen. Detailed investigation of TEM sections of the vertebral bone revealed presence of several electron dense spots of unknown origin in knock-outs. These “dark spots” may reflect extrafibrillar hydroxyapatite crystals (Figure 7B).

Mass spectrometry analysis of type I collagen modification in bone derived from the vertebral column showed no significant difference in hydroxylation status of the α1(I) K87 and C-telopeptide lysine residues (Supplementary Table S9). These lysine residues are conserved in zebrafish and human type I collagen and are crucial for intermolecular crosslinking of collagen fibrils in bone matrix (Gistelinck et al., 2017).

### *b3galt6* Knock-Out Zebrafish Display Ultrastructural Muscle Abnormalities and Lower Endurance

Human spEDS-*B3GALT6* patients often suffer from muscle hypotonia with a delay in motor development. In view of the observed reduction in the concentration of the different GAG moieties in muscle of *b3galt6*^–/–^ zebrafish, we investigated the function and the ultrastructural appearance of muscle.

**FIGURE 8:**
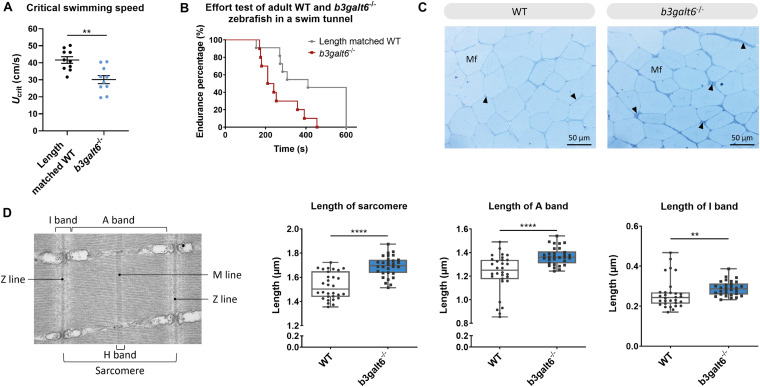
Functional and structural analysis of adult WT and *b3galt6*^–/–^ zebrafish muscle. **(A)** The critical swimming speed and **(B)** the endurance performance was determined for *b3galt6*^–/–^ and length-matched WT zebrafish. **(C)** Semi-thin cross sections, stained with toluidine blue, demonstrate the presence of a thicker endomysium (arrowhead), located between the muscle fibers (Mf) at the horizontal myoseptum of *b3galt6*^–/–^ zebrafish at the age of 5 months. **(D)** Transmission electron microscope structure of a sarcomere with indications of the Z and M line and A, I, and H band. The sarcomere length was determined at 5 different loci per semi-thin section, for 6 semi-thin sections origination from 3 different zebrafish per genotype (*N* = 30). A significant increase in sarcomere length for *b3galt6*^–/–^ is noted. Furthermore, both the A and I band show an increase in length for the mutant zebrafish. Data are expressed as box plots with min-to-max whiskers on which each individual data point is plotted. A Mann-Whitney U test was used to determine significance. ****P < 0.0001, **P < 0.01.

First, the critical swimming speed (*U*_*crit*_) and endurance of the zebrafish were assessed by means of a custom-made swim tunnel. The *U*_*crit*_, defined as the maximum velocity that the tested zebrafish could sustain for at least 5 min (Plaut, 2000), was significantly lower in *b3galt6*^–/–^ zebrafish (30.1 ± 2.39 cm/s) compared to length-matched WT (41.61 ± 1.942 cm/s) (*P* < 0.01) (Figure 8A). The stamina of each zebrafish was determined by an effort test where it was tested how long zebrafish could swim at high speed (37 cm/s) after a 3 min warm-up. This effort test showed that the *b3galt6*^–/–^ zebrafish performed less well compared to length-matched WT zebrafish, indicating a lower endurance (Figure 8B).

The muscle tissue in the zebrafish trunk was further investigated at the ultrastructural level. Toluidine blue-stained semi-thin cross sections revealed that the endomysium, a layer of connective tissue ensheating the muscle fibers, was enlarged along the horizontal myoseptum of *b3galt6*^–/–^ zebrafish, although no clear differences in muscle fiber diameter were noted (Figure 8C). Ultra-thin parasaggital sections, investigated by TEM, showed that the length of the sarcomeres, the smallest basic units of striated muscle, as well as the length of the A and I band were significantly increased in the *b3galt6*^–/–^ zebrafish (Figure 8D).

## Discussion

In this study we developed two viable *b3galt6* KO zebrafish models using CRISPR/Cas9 mutagenesis, and performed an in-depth characterization of the biochemical, ultrastructural and phenotypical consequences of β3GalT6-deficiency on different tissues, including skin, cartilage, mineralized bone and muscle. These models currently represent the only vertebrate models available for the study of the pathogenetic consequences of β3GalT6-deficiency on tissue development and homeostasis. In both models we detected a significant reduction, yet not a complete absence in *b3galt6* mRNA expression (Figure 1E). One possible explanation is the absence of canonical nonsense-mediated decay (NMD) in the single-exon *b3galt6* gene, due to a lack of exon junction complexes (EJCs) (Wittkopp et al., 2009). We were unable to examine the effect of the mutations on B3galt6 protein levels and localization due to the unavailability of a suitable antibody. Nevertheless, since the cmg20 and cmg22 alleles are predicted to introduce a PTC, respectively, upstream and in the catalytic domain, residual translation of some mRNA into a truncated protein is not expected to lead to the production of an active enzyme. β3galt6 is a key enzyme in the biosynthesis of the common linkage region of PGs, and reduced levels of active enzyme are thus expected to equally affect the biosynthesis of HS and CS/DS GAG chains. We therefore quantified the disaccharide concentrations in bone, muscle and skin of adult *b3galt6*^–/–^ zebrafish by means of a combination of enzymatic digestion and anion-exchange HPLC. This revealed a distinct, and relatively proportional decrease in the concentration of CS, DS, hybrid CS/DS and HS moieties in all examined tissues, compared to age-matched WT zebrafish (Table 1). Nonetheless, low amounts of GAGs were still produced in the KO zebrafish in absence of upregulation of other galactosyltransferase member(s) from the *b3galt* family or by other linkage-enzymes (Supplementary Figure S6). Together with the previous demonstration that the other five β3GalT human enzymes (e.g., β3GalT1, β3GalT2, β3GalT3/β3GalNAcT1, β3GalT4, and β3GalT5) are involved in the biosynthesis of glycoproteins and glycolipids (Bai et al., 2001), compensation for galactosyltransferase II deficiency in zebrafish by other *b3galt* family members is unlikely. Chinese hamster ovary *B3galt6* KO cells were also shown to be capable of producing GAGs, in contrast to *Xylt2*, *B4galt7* or *B3gat3* KO cells (Chen et al., 2018). The recent identification of the presence of a non-canonical trisaccharide linkage region, lacking one Gal residue (GlcA-Gal-Xyl-*O*-), as a minor constituent in the PG bikunin in the urine of healthy human individuals (Persson et al., 2019), prompted us to examine the composition of the GAG linkage region in our *b3galt6* KO zebrafish models, by analyzing protein extracts from adult *b3galt6*^–/–^ and WT zebrafish via LC-MS/MS. This led to the striking finding that the few detected PGs in the *b3galt6*^–/–^ zebrafish all presented with a trisaccharide PG linkage region, consisting of only one Gal (Figure 2B and Supplementary Table S6). Further *in vitro* analysis indicated that GlcAT-I/B3GAT3 is capable of adding a GlcA to Gal-Xyl-*O*- (Supplementary Figure S8). This observation might explain the phenotypic differences observed between GalT-II/B3GALT6 deficiency and deficiencies of the other linkage region enzymes, for which such a rescue mechanism might be absent. This hypothesis is supported by the lack of GAGs in the Chinese hamster ovary *Xylt2*, *B4galt7* or *B3gat3* KO cells and by the lethal phenotype observed in the *b4galt7* KO zebrafish model (Delbaere et al., 2020b). At present, we have no information on the length of the GAG chains attached to the trisaccharide linkage region or the composition and the possible modifications of these HS and CS/DS chains. As such, the GAG structure, length and modifications in the *b3galt6*^–/–^ zebrafish might be different from the ones in WT zebrafish.

HSPGs and CS/DSPGs are essential macromolecules for the development, signaling and homeostasis of many tissues. It therefore comes as no surprise that defects in linkage region biosynthesis can affect many organs. Endochondral ossification of cartilage (forming chondral bones) for example is a multi-staged process that must be coordinated in space and time. CS/DSPGs and HSPGS, which have key roles in modulating signaling events during vertebrate skeletal development, are expressed in a spatially and temporally highly controlled manner (Eames et al., 2011), thus regulating the timing of endochondral ossification. Genetic defects disrupting PG synthesis or structure have been shown to disrupt the timing of skeletal development. Two zebrafish models with abnormal PG linkage region biosynthesis, i.e., the *fam20b* and *xylt1*-deficient models, revealed partial decrease in CSPGs, which are predominantly present in cartilage, and accelerated endochondral ossification, showing that CSPGs can negatively regulate skeletogenic timing (Eames et al., 2011). On the other hand, a strong reduction in CSPGs and partial decrease in HSPGs was noted in *b3gat3* mutant zebrafish, but this led to a delay in endochondral ossification and short and disorganized cartilaginous structures (Holmborn et al., 2012). Our B3galt6-deficient zebrafish models also show a delay in skeletal development at juvenile stages. At 20 dpf, a delay was noticed in the development of cartilage and mineralized bony elements of the craniofacial structures, the pectoral and caudal fin endoskeleton (Figure 3B and Supplementary Tables S7, S8), and these abnormalities had progressed in adult zebrafish. The reduced body length, misshapen cranial bones and smaller teeth observed in the head skeleton, and general skeletal dysplasia with kyphosis and scoliosis seen in the vertebral column of *b3galt6*^–/–^ adult zebrafish is reminiscent of the skeletal phenotype observed in human β3GalT6-deficient individuals (Table 2).

In addition to skeletal dysplasia, we also observed abnormalities in bone mineralization. Adult mutant zebrafish stained with AR showed the presence of extra intramembranous bone and extra bony elements on the associated elements of the vertebral centra. On the other hand, quantitative μCT analysis of the vertebral column and associated elements revealed a generalized reduction in bone volume and thickness, and a relative increase in TMD after correction for reduced body length of the mutant zebrafish (Figure 6 and Supplementary Figure S13). Although it is not clear so far if the increased fracture risk in spEDS-*B3GALT6* patients can be attributed to a lower bone volume combined with a higher TMD, such hypothesis has been put forward for the brittle bone disorder Osteogenesis Imperfecta (OI) based on observations in both patients and animal models (Gistelinck et al., 2016; Fiedler et al., 2018). In the Chihuahua (*col1a1a*^*chi/+*^) OI zebrafish model the intrafibrillar mineral platelets were shown to be smaller and more densely packed, leading to an increased mineral to matrix ratio, and consequently a reduced elastic modulus-to-hardness ratio, a measure for fracture toughness (Viguet-Carrin et al., 2006; Bishop, 2016; Fiedler et al., 2018). In addition, in both OI patients and the mouse model *oim*, reduced collagen matrix organization was shown to lead to the formation of extrafibrillar crystals, which are larger compared to intrafibrillar crystals (Viguet-Carrin et al., 2006). Eventually, additional minerals stabilize the tissue, but they do so at the cost of increased brittleness of the bone (Grabner et al., 2001). In accordance, in *b3galt6*^–/–^ zebrafish we observed an irregular collagen organization interspersed with large interfibrillar spaces and electron dense spots on ultra-thin sections of skin and bone via TEM. The “dark spots” observed in the vertebral bone could be remnants of extrafibrillar mineral aggregates, contributing to increased mineralization but also increased brittleness of the skeleton.

Abnormalities in collagen fibril organization were observed in many mutant zebrafish tissues. Besides vertebral bone, irregular collagen organization was also observed in the intervertebral space, intervertebral ligament, and the dermis (Figure 7). This strongly resembles the previously reported abnormal dermal collagen fibril architecture in the skin from a β3GalT6-deficient individual (Malfait et al., 2013). This could be attributed to abnormalities in GAG chains of small leucine-rich PGs (SLRPs), such as the DSPGs decorin and biglycan, which are known to be of particular importance for regulating collagen fibril organization in connective tissues through their core protein and GAG chains (Kalamajski and Oldberg, 2010). SLRPs are also thought to influence collagen cross-linking patterns, but the mechanism by which they do so remain largely unknown (Kalamajski and Oldberg, 2010). Although similar hydroxylation levels were observed at K87 and the C-telopeptide of the type I collagen α1 chain in *b3galt6*^–/–^ and WT adult zebrafish (Supplementary Table S9), we cannot rule out that type I collagen cross-linking might still be affected.

In view of the observation that human β3GalT6-deficient individuals often present quite severe muscle hypotonia with delayed gross motor development and reduced endurance, we also studied the effect of B3galt6-deficiency on GAG synthesis in muscle, and on muscle function and ultrastructure (Figure 8). Similar to the other examined tissues, HS and CS/DS GAG concentrations were severely reduced in muscle tissue. It is known that the ECM in skeletal muscle plays an important role in muscle fiber force transmission, maintenance and repair. Numerous PGs, including the HSPGs syndecans 1–4, glypican-1, perlecan and agrin, and CS/DSPGs, such as decorin and biglycan, are present in muscle ECM. Besides their structural role, many of them are known to bind growth factors (e.g., TGFβ). Those growth factors can be stored and released by the negatively charged GAGs. Certain enzymes in the ECM can cleave GAG chains, thereby releasing associated growth factors, allowing their interaction in cell signaling and mechanotransduction (Gillies and Lieber, 2011). The significant decrease in critical swimming speed (*U*_*crit*_) and endurance observed in the *b3galt6*^–/–^ zebrafish suggest decreased muscle function (Figures 8A,B). Further evidence for an intrinsic muscle dysfunction in the *b3galt6*^–/–^ model comes from semi-thin muscle sections, showing an enlargement of the connective tissue sheet surrounding the muscle fibers (Figure 8C). Nevertheless, we cannot exclude that the observed skeletal malformations and/or other abnormalities, such as respiratory or neuronal dysfunction, could also influence the outcome of these tests.

Taken together, we generated and characterized the first *b3galt6* KO zebrafish models and show that their phenotype largely mimics that of human β3Galt6-deficiency. A strong reduction in HS, CS and DS GAG concentration was observed in *b3galt6*^–/–^ zebrafish. Yet, we did observe a small amount of GAGs being produced with a unique trisaccharide PG linkage region. Furthermore, we showed that a lack of B3galt6 causes disturbances in collagen fibril organization in skin, ligament and bone, with the latter also showing abnormalities in mineralization, and leads to structural and functional abnormalities of the muscle. These KO zebrafish can serve as useful models to further unravel the underlying pathogenic mechanisms of this complex multisystemic disorder and serve as a tool for the development and evaluation of possible therapeutic interventions, such as the stimulation of intrinsic compensational mechanisms for the production of PGs.

Future studies will focus on verifying the presence of a trisaccharide linkage region in samples (e.g., urine) from human spEDS-*B3GALT6* patients. In addition, the hypothesis that B3gat3 is responsible for the remaining GAG synthesis in our *b3galt6*^–/–^ model should be further explored by inactivating B3gat3 in our *b3galt6*^–/–^ model via knock-down or knock-out approaches, followed by GAG analysis. Potential therapeutic avenues to explore include stimulation of B3gat3 expression, or enzyme replacement therapy (ERT). For patients with an inborn error of metabolism, such as Gaucher disease and mucopolysaccharidosis, ERT is a well-known therapeutic approach and has already been proven successful (Ferreira and Gahl, 2017). Other (but less straightforward) therapeutic avenues in human are stimulation of residual enzyme via co-enzyme treatment or enzyme enhancement therapy, bone-marrow transplantation or correcting the product deficiency by providing alternate substrate. Nevertheless, we should first investigate the human situation before starting to explore these avenues.

## Materials and Methods

### Zebrafish Maintenance

Wild-type (AB strain) and *b3galt6* knock-out zebrafish lines were reared and maintained by standard protocols (Gistelinck et al., 2018). All animal studies were performed in agreement with EU Directive 2010/63/EU for animals, permit number: ECD 16/18. All zebrafish used in a single experiment were bred at the same density. All efforts were made to minimize pain, distress and discomfort.

### Alcian Blue and Alizarin Red Staining

Twenty-day-old larvae (*n* = 10) and 5 months old WT and *b3galt6*^–^*^/^*^–^ (*n* = 5) adult zebrafish were stained as previously described (Delbaere et al., 2020b). See Supplementary Information for details.

### μCT Scanning Analysis

Whole-body μCT scans of 4 months old WT (*n* = 5) and *b3galt6*^–/–^ (*n* = 5) siblings were acquired on a SkyScan 1275 (Bruker, Kontich, Belgium) using the following scan parameters: 0.25 mm aluminum filter, 50 kV, 160 μA, 65 ms integration time, 0.5° rotation step, 721 projections/360°, 5 m 57 s scan duration and 21 μm voxel size. DICOM files of individual zebrafish were segmented in MATLAB using custom FishCuT software and data were analyzed and corrected for standard length in the R statistical environment, as previously described (Hur et al., 2017).

### Histology and Transmission Electron Microscopy

Zebrafish bone, consisting of the last transitional vertebra and two consecutive caudal vertebrae (Morin-Kensicki et al., 2002; Bird and Mabee, 2003), skin, at the lateral line below the dorsal fin, and muscle samples, located dorsolateral underneath the dorsal fin, dissected from 5 months old WT (*n* = 5) and *b3galt6*^–/–^ (*n* = 5) zebrafish, were fixed and embedded in epon following the procedures outlined by Huysseune and Sire (1992).

### Swim Tunnel Experiments

Ten WT (5 months) and ten *b3galt6*^–^*^/^*^–^ zebrafish (8 months), were selected based on their similar (not significantly different) length and weight. The critical swimming speed (*U*_*crit*_) was measured in a swim tunnel for both WT and *b3galt6*^–/–^ zebrafish as previously described (Brett, 1964). Furthermore, the endurance of each zebrafish was tested by means of an effort test in the swim tunnel. Each zebrafish had a 3 min warm-up and after the third minute, the speed increased until 37 cm/s for maximum 7 min. When the zebrafish was not able to swim anymore, the test was stopped.

### Collagen Crosslinking Analysis

The collagen crosslinking was analyzed in the dissected vertebral column (excluding Weberian apparatus and urostyle) from 5 months old wild-type (*n* = 5) and *b3galt6*^–/–^ (*n* = 5) siblings as described before in Gistelinck et al. (2016).

### Disaccharide Composition Analysis of CS, DS, and HS Chains

The level of total disaccharides from CS, DS, and HS in bone, muscle and skin tissues from 9 months old zebrafish (*n* = 5) were determined as described previously (Mizumoto et al., 2009; Mizumoto and Sugahara, 2012).

### Glycopeptide Preparation and Analysis

#### LC-MS/MS Sample Preparation

CS- and HS-glycopeptides were purified from 6 months old WT (*n* = 3) and *b3galt6*^–/–^ (*n* = 3) zebrafish using a combination of extraction, trypsin digestion and anion exchange chromatography, slightly modified (see Supplementary Information) from previously described protocols (Noborn et al., 2015, 2016).

#### LC-MS/MS Analysis

The samples were analyzed on an Orbitrap Fusion mass spectrometer coupled to an Easy-nLC 1200 liquid chromatography system (Thermo Fisher Scientific., Waltham, MA) (see Supplementary Information). Nanospray Flex ion source was operated in positive ionization mode and three separate higher-energy collision-induced dissociation (HCD) MS/MS spectra were recorded for each precursor ion (Noborn et al., 2015, 2016).

#### LC-MS/MS Data Analysis

The glycopeptide files were analyzed using both manual interpretation and automated Mascot searches for GAG-glycopeptides. The files were manually interpreted using the Xcalibur software (Thermo Fisher Scientific). Database searches were performed against *Danio rerio* in the UniProtKB/Swiss-Prot database and NCBI using Mascot Distiller (version 2.6.1.0, Matrix Science, London, United Kingdom) and an in-house Mascot server (version 2.5.1). Glycan structures with very small mass differences were manually evaluated at the MS2-level.

#### GlcAT-I Assay

GlcA-transferase assay was carried out by the methodology as previously described (Kitagawa et al., 1998; Baasanjav et al., 2011; Job et al., 2016), with slight modifications.

Detailed information regarding the procedures used is provided in Supplementary Material and Methods.

## Data Availability Statement

The original contributions presented in the study are included in the article/Supplementary Material, further inquiries can be directed to the corresponding author.

## Ethics Statement

The animal study was reviewed and approved by the Ethische Commissie Dierproeven Faculteit Geneeskunde en Gezondheidswetenschappen Universiteit Gent. All animal studies were performed in agreement with EU Directive 2010/63/EU for animals, permit number: ECD 16/18.

## Author Contributions

SD, AW, and FM designed the study and wrote the manuscript. SD, AD, SM, FN, JB, LA, and CG performed the research. SM, PS, GL, and SY contributed new reagents and analytic tools. SD, AD, SM, FN, JB, LA, CG, DS, PS, PC, GL, SY, AW, and FM analyzed the data. All authors reviewed the manuscript.

## Conflict of Interest

PS was employed by the company Bruker MicroCT. The remaining authors declare that the research was conducted in the absence of any commercial or financial relationships that could be construed as a potential conflict of interest.
